# Diabetes-Associated Focal Myonecrosis: A Case Report and Literature Review

**DOI:** 10.7759/cureus.65323

**Published:** 2024-07-25

**Authors:** Hafiz Muhammad Hassan Shoukat, Namra Ajmal

**Affiliations:** 1 Internal Medicine, Cooper University Hospital, Camden, USA; 2 Pathology, Thomas Jefferson University Hospital, Philadelphia, USA

**Keywords:** rare complication, hba1c, uncontrolled diabetes, muscle infarction, focal myonecrosis

## Abstract

Diabetes-associated focal myonecrosis is a rare complication seen in individuals with long-standing uncontrolled diabetes, characterized by inflammation and necrosis of a single or group of muscles. The exact cause of this condition is not well understood, but it is believed to be due to focal muscle infarction secondary to arteriosclerosis and diabetic microangiopathy. Diagnosis is challenging and often requires clinical examination, lab investigations, imaging, and EMG. Treatment is mainly supportive with pain control and tight glycemic control, and surgical intervention is rarely needed. The clinical presentation includes a sudden onset of localized pain and swelling in the affected muscle, which may be accompanied by fever, malaise, and weight loss. Diabetic myonecrosis exhibits a slightly higher prevalence in females and commonly manifests at an early stage. While the short-term prognosis is good, the recurrence rate is high, often affecting the opposite limb within six months. Our case describes a 35-year-old young male with uncontrolled diabetes mellitus, diagnosed one year ago, who presented with medial thigh pain and tenderness for the last two days. Due to his early disease, focal myonecrosis was not our first differential diagnosis. A CT scan with contrast revealed findings consistent with either focal myositis or infarction. We ruled out other causes, including infections, autoimmune disease, trauma, and medications, and in combination with the patient’s uncontrolled diabetes mellites, a diagnosis of diabetes-associated focal myonecrosis was made. The patient improved with blood sugar control and supportive care, including nonsteroidal anti-inflammatory drugs and muscle relaxants.

## Introduction

Diabetes-associated focal myonecrosis is a rare complication characterized by localized inflammation and necrosis of a single muscle or a group of muscles and is usually seen in long-standing, uncontrolled diabetes. Diabetic myonecrosis (DMN) is a rare disease with an unclear incidence, as evidenced by the limited number of cases documented in the literature to date, numbering only in the hundreds. It was initially described in 1965 by Angervall and Sterner. The exact etiology of diabetes-associated focal myositis/myonecrosis is not yet fully understood. However, it is believed to be due to focal muscle infarction secondary to arteriosclerosis and diabetic microangiopathy [1,2]. Clinical exams, lab investigations, imaging, and electromyography usually make the diagnosis. A definitive diagnosis requires a muscle biopsy, which is generally unnecessary and reserved for atypical presentations. Diabetic muscle infarction or myonecrosis usually resolves spontaneously, but recurrence has been reported in 50% of the patients [1].

## Case presentation

A 35-year-old African American male presented with a sudden onset of left medial thigh pain and tenderness over the last two days, causing marked discomfort and difficulty ambulating. He had a significant past medical history of type 2 diabetes mellitus and essential hypertension, diagnosed nearly one year prior when he was admitted to the hospital with severe diabetic ketoacidosis (DKA). He was discharged on subcutaneous insulin and antihypertensive medications but lost follow-up with his primary care physician (PCP) and discontinued his medications. The patient reported daily tobacco smoking, approximately half a pack per day, and occasional alcohol use.

In the emergency room, the patient was afebrile, with a blood pressure of 190/75 mmHg, a heart rate of 110 bpm, and an oxygen saturation of 96% on room air. Physical examination was otherwise benign except for a cord-like structure in the left medial thigh, which was extremely tender on palpation but had no overlying redness or erythema. The patient also complained of polyuria but denied any fever, chills, cough, chest pain, nausea, vomiting, diarrhea, dysuria, skin rash, arthralgia, or myalgias. There was no recent viral infection, and the patient was not taking any medication at home.

Routine laboratory investigations revealed the following: WBC was 8.8 K/µL (normal range: 3.7-11.0 K/µL), hemoglobin 14.4 g/dL, platelet count 460 K/µL, creatine kinase 331 U/L (normal range: 42-196 U/L), CRP 50 mg/L, creatinine 0.8 mg/dL, blood urea nitrogen 10 mg/dL, bicarbonate 22 mEq/L, anion gap 12, potassium 4 mEq/L, sodium 131 mEq/L, glucose 429 mg/dL, calcium 9.5 mg/dL. Liver function tests were normal, and urinalysis showed no evidence of a UTI. No evidence of DKA was noted based on these investigations.

**Figure 1:**
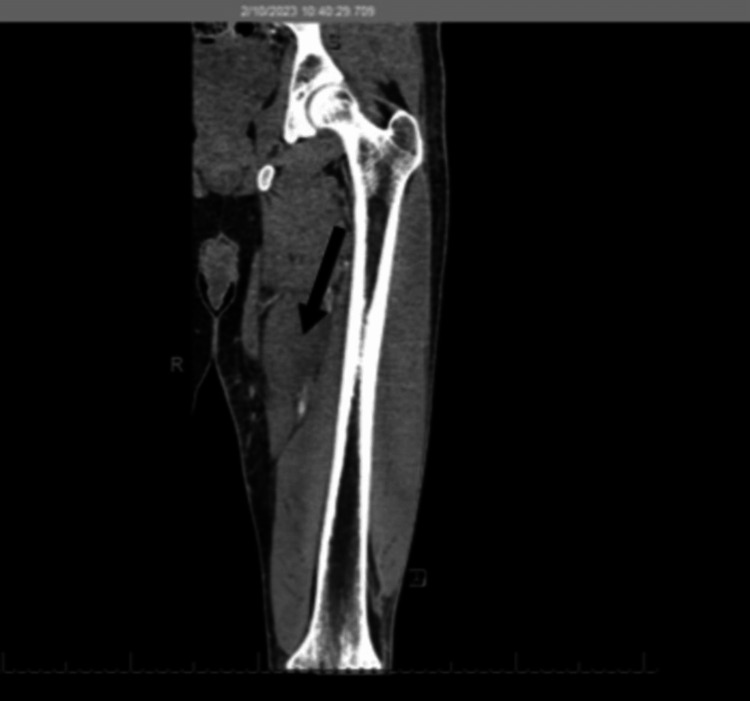
Coronal CT of the left lower extremity showing hypoattenuation of the adductor longus muscle within the medial compartment and associated subcutaneous edema (arrow)

**Figure 2:**
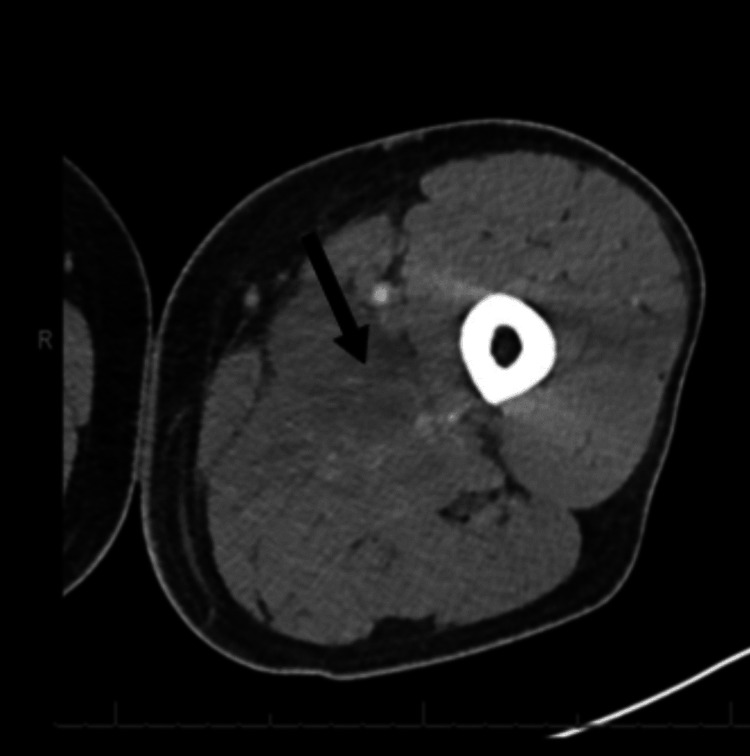
Transverse CT of the left lower extremity showing hypoattenuation of the adductor longus muscle within the medial compartment (arrow)

The patient underwent a left lower extremity CT scan with intravenous contrast, which revealed focal hypoattenuation of the adductor longus muscle. A small volume of fluid was noted in the subcutaneous tissue surrounding the medial thigh compartment, consistent with myositis (black bold arrow in Figure 1 and Figure 2). No drainable fluid or abscess was identified.

The patient was initially started on IV fluid resuscitation and subcutaneous insulin. Due to the unclear etiology, blood cultures were taken, and broad-spectrum IV antibiotics, including Zosyn and vancomycin, were administered in the emergency room. Further antibiotics were discontinued as there was no convincing evidence of sepsis or infectious etiology. Blood cultures and MRSA nasopharyngeal swab screens remained negative, and the antinuclear antibody screen was negative (<1:40).

The patient’s blood glucose levels improved with IV hydration and subcutaneous insulin. Hemoglobin A1C was 17.8, indicating poorly controlled hyperglycemia. The pain was successfully managed with nonsteroidal anti-inflammatory drugs (NSAIDs) and muscle relaxants, and the patient began ambulating with minimal difficulty. The subcutaneous insulin regimen was adjusted, and lisinopril was added for better blood pressure control. Diabetic education was provided, and the patient was advised on smoking cessation, medication compliance, and the importance of follow-up with the PCP.

## Discussion

DMN was first described by Angervall and Sterner in 1965 as “tumoriform focal muscular degeneration” [2]. It is usually considered a complication of long-standing uncontrolled diabetes mellites, and diagnosis is often challenging as it can mimic other conditions, such as deep vein thrombosis, cellulitis, or soft tissue tumors.

The exact etiology of DMN is not well understood. However, it is thought to be related to microangiopathy secondary to long-standing uncontrolled diabetes. An alternative hypothesis regarding the development of DMN suggests the existence of hypercoagulability, including antithrombin II deficiency, elevated levels of factor VII, hyperhomocysteinemia, the presence of antiphospholipid antibodies, and reduced levels of both prostacyclin and tissue plasminogen activator. These factors may culminate in the occurrence of muscle infarction [3].

The clinical presentation includes a sudden onset of localized pain and swelling in the affected muscle (calf and thigh muscles are typically involved), which may be accompanied by fever, malaise, and weight loss. Laboratory tests may reveal elevated creatine kinase levels, CRP, and erythrocyte sedimentation rate. Imaging studies, such as an MRI or CT scan with contrast, can help confirm the diagnosis by showing characteristic findings of muscle edema, necrosis, and inflammation. A muscle biopsy is rarely needed in cases of atypical presentations. Treatment is mainly supportive of pain control and tight glycemic control [2,3]. Some experts suggest low-dose aspirin due to its antithrombotic effects, but no sufficient evidence of its benefits has been studied yet. Hospitalization and intravenous fluids are needed if there is significant myolysis, and a fasciotomy may be required to prevent or treat compartment syndrome. Surgical intervention is usually not needed, and the mean duration of recuperation for individuals receiving aspirin and/or NSAIDs was found to be approximately 5.5 weeks [2].

DMN exhibits a slightly higher prevalence in females and commonly manifests at an early stage. Some studies suggest the average duration of diabetes at the point of diagnosis was approximately 18.9 years for type 1 diabetes and 11 years for type 2 diabetes. The average HbA1c level at diagnosis was approximately 9.34% [3,4]. Although a short-term good prognosis was noted, a high recurrence rate was noted, ranging from 34.9% to 47.8%, often affecting the opposite limb within six months [3].

## Conclusions

Diabetes-associated focal myonecrosis poses a diagnostic challenge, especially in early disease, as these complications are expected to be seen in long-standing diabetes mellitus, but it can present as an early disease, as in our case. MRI or CT with contrast usually shows a similar picture, with a typical presentation, a typical location in thigh or calf muscles, and imaging consistent with focal myonecrosis, which can help to make a diagnosis with confidence and to avoid invasive investigations like muscle biopsy. Tight glycemic control and regular follow-up are critical to observing the resolution of symptoms and preventing a recurrence.
